# Association Between Dietary Fatty Acid Intake With Cardiovascular Disease Risk and Serum Lipid Levels

**DOI:** 10.1002/edm2.70148

**Published:** 2025-12-21

**Authors:** Naeemeh Hassanpour Ardekanizadeh, Khadijeh Abbasi Mobarakeh, Sheyda Nami, Maryam Shojaei, Seyed Fazel Nasimi, Saba Moradi, Faezeh Tejareh, Yeganeh Shekari, Parsa Bahmani, Maryam Gholamalizadeh, Marjan Ajami, Akram Kooshki, Saeid Doaei

**Affiliations:** ^1^ Department of Clinical Nutrition, School of Nutrition and Food Sciences Shiraz University of Medical Sciences Shiraz Iran; ^2^ Food Security Research Center and Department of Community Nutrition, School of Nutrition and Food Science Isfahan University of Medical Sciences Isfahan Iran; ^3^ Iran University of Medical Sciences Tehran Iran; ^4^ Central Research Laboratory, School of Medicine Shiraz University of Medical Sciences Shiraz Iran; ^5^ Master of Science in Nutrition and Exercise Physiology Zand Institute of Higher Education Shiraz Iran; ^6^ Departman of Medicine Qazvin University of Medical Sciences Qazvin Iran; ^7^ Department of Nutrition, Science and Research Branch Islamic Azad University, Tehran Medical Branch Tehran Iran; ^8^ Department of Nutrition, Faculty of Medical Sciences, Science and Research Branch Islamic Azad University Tehran Iran; ^9^ Department of Community Nutrition and Dietetics, Faculty of Nutrition and Food Technology Shahid Beheshti University of Medical Sciences Tehran Iran; ^10^ Unit of Nutrition and Cancer, Cancer Epidemiology Research Program Catalan Institute of Oncology Bellvitge Biomedical Research Institute (IDIBELL), L'Hospitalet de Llobregat Barcelona Spain; ^11^ Cancer Research Center Shahid Beheshti University of Medical Sciences Tehran Iran; ^12^ Department of Food and Nutrition Policy and Planning, School of Nutrition Sciences and Food Technology, National Nutrition and Food Technology Research Institute Shahid Beheshti University of Medical Sciences Tehran Iran; ^13^ Non‐Communicable Diseases Research Center Sabzevar University of Medical Sciences Sabzevar Iran

**Keywords:** cardiovascular disease, dietary fats, lipid profile, monounsaturated fatty acids

## Abstract

**Background:**

Despite early interest in the effects of dietary fats on cardiovascular diseases (CVDs), substantial controversy remains regarding the evidence linking different types of fatty acids to CVDs. This study aimed to examine the association between dietary fat intake, CVD risk, and serum lipid biomarkers.

**Methods:**

This cross‐sectional study included data from 4200 adult participants (1218 patients with CVDs and 2982 healthy participants) from the Persian Cohort Study. Data on heart disease (hypertension, myocardial infarction, and ischemic heart disease) were collected. Dietary intake was assessed using a validated food frequency questionnaire (FFQ), and the intake of different fatty acids was evaluated using Nutritionist‐IV software. Serum lipid profiles were analysed using enzymatic and chromatographic methods.

**Results:**

Higher monounsaturated fatty acids (MUFA) intake showed an inverse association with CVDs (OR = 0.931, 95% CI: 0.867–0.998, *p* = 0.045). Adjustments for age, gender, smoking, alcohol consumption, physical activity, BMI, and caloric intake did not alter this association. No significant associations were observed for other dietary fats.

**Conclusion:**

The findings suggest an inverse association between MUFA intake and CVD risk. Further longitudinal studies are warranted to confirm these results.

## Graphical abstract

The findings suggest a protective role of MUFA intake against CVDs risk.

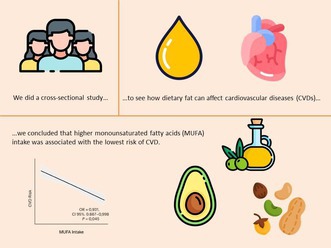

## 1 Introduction

Cardiovascular diseases (CVDs) are the leading cause of global mortality, accounting for an estimated 17.9 million deaths annually [1]. About four out of five CVD deaths are due to heart attacks and strokes [1]. CVDs are responsible for 46% of all deaths and 20% of the burden of disease in Iran [2]. The main cardiometabolic risk factors of CVDs are diabetes, hypertension, dyslipidemia, and excess abdominal fat, which are all affected by dietary intake [3].

Dietary components can potentially influence the initiation and progression of CVDs [4]. Among them, dietary fats are reported to be key contributors to the development of CVDs and are associated with morbidity and mortality [5]. Although there is still disagreement about the effect of saturated fatty acids (SFA) and trans fatty acids (TFA) on CVDs, many studies emphasised the adverse effect of these fatty acids on cardiovascular function [6, 7]. On the other hand, a reduction in the risk of cardiovascular problems has been observed with increasing polyunsaturated fatty acids (PUFA) intake [8]. This effect can be due to their role in increasing membrane mobility and flexibility [9].

Moreover, an inflammatory state can increase the risk of developing chronic diseases such as CVDs [10]. Systemic and local inflammation has a central role in the development and progression of CVDs, from endothelial dysfunction to clinical symptoms [11]. Inflammatory biomarkers have been shown to predict CVDs independently of traditional risk factors [12]. Higher levels of omega‐3 fatty acids, an anti‐inflammatory factor, were significantly associated with reduced overall CVDs risks [13, 14]. Also, the essential omega‐6 PUFA, linoleic acid (LA), is suggested to decrease the risk for CVDs by affecting the lipid‐related risk factors [15]. On the other hand, extensive lines of evidence imply a disease‐specific contribution of inflammation‐related metabolites of arachidonic acid (AA) to CVDs and their complications [8, 16, 17]. Although this effect of AA has not been confirmed in some other studies [13]. Modifying the ratio of omega‐3 and omega‐6 intake is considered a strategy for dietary recommendations [14, 18, 19, 20]. The cardiovascular effects of different types of fatty acids remained controversial [13]. In this article, despite the existing contradictions, we intend to help improve nutritional recommendations by examining the association of different types of fats in the diet with improving CVDs and the levels of their serum biomarkers.

## 2 Methods

### 2.1 Population and Data

This study was a cross‐sectional study using data from the Persian Cohort Study [21]. In this study, 4241 people were eligible for the study, all of whom were included in the study. Inclusion criteria was volunteering for the study, written informed consent, and age from 35 to 70 years. Exclusion criteria included unwillingness to participate or continue cooperation, inability to attend physical examinations or complete questionnaires, diagnosis of severe psychiatric disorders or cognitive impairment interfering with data collection, current use of medications affecting lipid metabolism (e.g., corticosteroids, statins, barbiturates, carbamazepine), presence of conditions such as liver disease, diabetes, or neurological disorders that could confound lipid profiles, and incomplete or missing medical records. Participants with hemolyzed blood samples that could distort serum lipid measurements were also excluded. Of the initial 4241 participants, 41 individuals were excluded based on predefined criteria, and the final analysis was conducted on 4200 participants. Cardiovascular disease (CVD) was defined as a composite outcome including self‐reported history of physician‐diagnosed hypertension, myocardial infarction (MI), and ischemic heart disease (IHD), as recorded in the Persian Cohort Study medical questionnaire. Cardiovascular disease (CVD) was diagnosed based on self‐reported physician diagnosis, hospitalisation records, and/or use of cardiovascular medications, consistent with previous cohort studies. Initially, patients' information included demographic characteristics (age, sex, marital status, and education), living, history of smoking, and history of alcohol consumption was extracted. The height and weight of the participants were measured using a standard calibrated Seca scale with an accuracy of 0.5 cm and 100 g by trained researchers, and then BMI was calculated by dividing weight (kg) by the square of height (meters).

### 2.2 Dietary Intake Assessment

To evaluate dietary‐related information, the data obtained from the Persian Cohort Food Frequency Questionnaire (FFQ), whose validity and reliability were previously confirmed [22], were converted into nutrients using Nutritionist‐IV software, and the intake of different types of dietary fats was estimated. The validation studies demonstrated acceptable reliability and moderate to strong correlations with multiple 24‐h recalls and nutrient biomarkers (e.g., serum lipids and vitamins). Data were collected by trained interviewers and standardised procedures. Different types of dietary fats were assessed including total fat, cholesterol, saturated fatty acids (SFA), trans fatty acids (TFA), monounsaturated fatty acids (MUFA), polyunsaturated fatty acids (PUFA), omega‐3 fatty acids [including alpha‐linolenic acid (ALA), eicosapentaenoic acid (EPA), and docosahexaenoic acid (DHA)], and omega‐6 fatty acids.

### 2.3 Serum Lipid Measurement

Blood samples were collected from participants after a minimum fasting period of 8 h. Specimens were drawn into sterile microcentrifuge tubes. Blood samples were centrifuged at 3000 RPM for 10 min at 4°C to separate serum from cellular components. The serum was then aliquoted and stored at −80°C for analysis. Lipid profiles were analysed using enzymatic methods employed for quantifying total cholesterol and triglycerides, involving specific enzymatic reactions that convert lipids into measurable products, and colorimetric assays utilised for various lipid measurements based on colour change resulting from chemical reactions. High‐Performance Liquid Chromatography (HPLC) was then applied for detailed analysis of lipid subclasses, ensuring high specificity and sensitivity. The measured lipid biomarkers included total cholesterol, triglycerides, low‐density lipoprotein cholesterol (LDL‐C), and high‐density lipoprotein cholesterol (HDL‐C).

### 2.4 Data Analyses

Descriptive statistics including mean and standard deviation were calculated to summarise demographic, social, and anthropometric characteristics. Independent T‐tests were applied to compare groups under the assumption of normal distribution, which was assessed using visual inspection and statistical tests where appropriate. To reduce random reporting error, we performed energy adjustment of nutrient intakes using the residual method. Moreover, sensitivity analyses excluding implausible energy reporters (< s800 or > 4200 kcal/day) yielded similar findings, suggesting the robustness of our results to potential misreporting. A binary logistic regression method was used to investigate the relationship between fat intake (as a continuous variable) and the presence of cardiovascular disease (CVD) as a dichotomous outcome variable. For continuous variables (age, BMI, physical activity, total energy intake, and serum lipid levels), we assessed linearity in the logit using the Box–Tidwell transformation and visual inspection of partial residual plots. All variables demonstrated approximately linear relationships with the log odds of CVD and were therefore modelled as linear terms. Potential confounders, including BMI, diabetes mellitus, and physical activity level, were evaluated and adjusted for in the regression models. All statistical analyses were performed using SPSS software version 27 (IBM Corp., Armonk, NY, USA). A two‐tailed *p*‐value of less than 0.05 was considered statistically significant in all analyses.

## 3 Results

Table 1 presents data on the distribution of characteristics of the participants. The patients had a higher mean age (54.19 ± 8.03 vs. 47.20 ± 8.23 y, *p* < 0.001), weight (79.98 ± 13.52 vs. 73.34 ± 13.35 kg, *p* < 0.001), BMI (29.38 ± 4.77 vs. 27.70 ± 4.62 kg/m^2^, *p* < 0.001), systolic blood pressure (124.59 ± 19.13 vs. 110.36 ± 14.16 mmHg, *p* < 0.001), diastolic blood pressure (77.40 ± 11.39 vs. 69.73 ± 9.29 mmHg, *p* < 0.001), cholesterol (192.54 ± 38.88 vs. 189.65 ± 43.26 mg/dL, *p* = 0.044), low‐density lipoprotein (111.92 ± 32.72 vs. 106.41 ± 35.69 mg/dL, *p* < 0.001), triglyceride (157.04 ± 92.84 vs. 142.09 ± 105.71 mg/dL, *p* < 0.001), and diabetes (26.7 vs. 8.9%, *p* < 0.001) than the controls. On the other hand, the healthy participants had a higher physical activity (39.8 ± 7.99 vs. 37.55 ± 7.17 kcal/kg/d, *p* < 0.001), height (162.76 ± 9.19 vs. 160.84 ± 9.14 cm, *p* < 0.001), job (47.1% vs. 30.5%, *p* < 0.001), and smoking (51.6% vs. 36.7%, *p* < 0.001) than the cases. Also, the proportion of female participants was significantly higher among CVDs cases compared to controls (46.1% vs. 40.8%, *p* < 0.002).

**TABLE 1:** Characteristics of the participants.

	Patients with CVDs (*n* = 1218)	Participants without CVDs (*n* = 2982)	*p*
Age (y)	54.19 ± 8.03	47.20 ± 8.23	< 0.001
Physical activity (kcal/kg/d)	37.55 ± 7.17	39.08 ± 7.99	< 0.001
Height (cm)	160.84 ± 9.14	162.76 ± 9.19	< 0.001
Weight (kg)	75.98 ± 13.52	73.34 ± 13.35	< 0.001
BMI (kg/m^2^)	29.38 ± 4.77	27.70 ± 4.62	< 0.001
Right SBP (mmHg)	124.59 ± 19.13	110.36 ± 14.16	< 0.001
Right DBP (mmHg)	77.40 ± 11.39	69.73 ± 9.29	< 0.001
TG (mg/dL)	157.04 ± 92.84	142.09 ± 105.71	< 0.001
CHOL (mg/dL)	192.54 ± 38.88	189.65 ± 43.26	0.044
HDL (mg/dL)	52.11 ± 10.84	52.50 ± 10.56	0.283
LDL (mg/dL)	111.92 ± 32.72	106.41 ± 35.69	< 0.001
Use alcohol (yes) (*n*, %)	79 (6.5%)	214 (7.2%)	0.463
Has diabetes (yes) (*n*, %)	325 (26.7%)	264 (8.9%)	< 0.001
Has job (yes) (*n*, %)	371 (30.5%)	1405 (47.1%)	< 0.001
Smoking (yes) (*n*, %)	77 (36.7%)	227 (51.6%)	< 0.001
Males (*n*, %)	497 (40.8%)	1374 (46.1%)	< 0.002
Females (*n*, %)	721 (59.2%)	1608 (53.9%)	< 0.002

Abbreviations: BMI, body mass index; CHOL, cholesterol; DBP1, diastolic blood pressure 1; HDL, high‐density lipoprotein; LDL, low‐density lipoprotein; SBP1, systolic blood pressure 1; TG, triglyceride.

Table 2 presents participants' dietary intake, comparing patients with CVDs (*n* = 1211) and individuals without CVDs (*n* = 2958). Patients had significantly lower daily intake of protein (74.8 ± 25.44 vs. 80.24 ± 25.07 g, *p* < 0.001), total fat (61.54 ± 25.68 vs. 65.72 ± 138.31 g, *p* < 0.001), carbohydrates (397.46 ± 136.34 vs. 418.99 ± 783.54 g, *p* < 0.001), energy (2392.61 ± 779.02 vs. 2541.04 ± 14.9 kcal, *p* < 0.001) compared to controls. Regarding dietary fats, patients had significantly lower intake of cholesterol (237.25 ± 110.16 vs. 268.88 ± 0.26 mg, *p* = 0.002), total trans fatty acids (0.22 ± 0.28 vs. 0.25 ± 11.30 g, *p* < 0.001), SFA (23.94 ± 11.28 vs. 25.64 ± 8.22 g, *p* < 0.001), MUFA (19.21 ± 8.53 vs. 20.46 ± 4.78 g, *p* < 0.001), PUFA (11.27 ± 4.954 vs. 11.98 ± 0.02 g, *p* < 0.001), and omega‐6 fatty acids (4.64 ± 3.59 vs. 4.91 ± 3.99 g, *p* < 0.001). However, no significant differences were observed in the intake of total omega‐3 fatty acids (*p* = 0.461) or eicosapentaenoic acid (EPA) (*p* = 0.093). Docosahexaenoic acid (DHA) intake was significantly lower in cases (0.01 ± 0.01 vs. 0.03 ± 0.01 g, *p* = 0.038), while alpha‐linolenic acid (ALA) intake was slightly higher in cases (6.77 ± 4.50 vs. 6.52 ± 3.28 g, *p* = 0.026).

**TABLE 2:** Dietary intake of the participants.

	Patients with CVDs (*n* = 1218)	Participants without CVDs (*n* = 2982)	P
Protein (g/d)	74.8 ± 25.44	80.24 ± 25.07	< 0.001
Total fat (g/d)	61.54 ± 25.68	65.72 ± 138.31	< 0.001
Carbohydrate (g/d)	397.46 ± 136.34	418.99 ± 783.54	< 0.001
Energy (kcal/d)	2392.61 ± 779.02	2541.04 ± 14.9	< 0.001
Cholesterol (mg/d)	237.25 ± 110.16	268.88 ± 0.26	0.002
Trans fatty acid (g/d)	0.22 ± 0.28	0.25 ± 11.30	< 0.001
Saturated fatty acid (g/d)	23.94 ± 11.28	25.64 ± 8.22	< 0.001
MUFA (g/d)	19.21 ± 8.53	20.46 ± 4.78	< 0.001
PUFA (g/d)	11.27 ± 4.954	11.98 ± 0.02	< 0.001
Omega‐3 fatty acid (g/d)	0.03 ± 0.02	0.03 ± 0.01	0.461
Docosahexaenoic acid (g/d)	0.01 ± 0.01	0.03 ± 0.01	0.038
Eicosapentaenoic acid (g/d)	0.46 ± 0.40	0.48 ± 4.03	0.093
Alpha‐linolenic acid (g/d)	6.77 ± 4.50	6.52 ± 3.28	0.026
Omega‐6 fatty acid (g/d)	4.64 ± 3.59	4.91 ± 3.99	< 0.001

Abbreviations: MUFA, monounsaturated fatty acid; PUFA, polyunsaturated fatty acid.

Regarding the association of CVDs with the lipid profile of the participants, significant positive associations were found between CVDs and serum level of total cholesterol (OR = 1.009, 95% CI:1.004–1.013, *p* < 0.001) and LDL (OR = 1.008, 95% CI:1.003–1.013, *p* = 0.001) (Table 3).

**TABLE 3:** The association of CVDs and the level of lipid profile biomarkers.

	Model 1		Model 2		Model 3		Model 4	
	OR^a^ (95% CI)	*p*	OR^a^ (95% CI)	*p*	OR^a^ (95% CI)	*p*	OR^a^ (95% CI)	*p*
Cholesterol	1.009 (1.004–1.013)	< 0.001	1.007 (1.003–1.012)	0.001	1.008 (1.003–1.013)	0.001	1.008 (1.004–1.013)	< 0.001
TG	1.000 (0.998–1.001)	0.675	0.999 (0.997–1.001)	0.357	1.000 (0.998–1.001)	0.739	1.000 (0.998–1.002)	0.773
HDL	1.011 (0.991–1.031)	0.286	1.017 (0.996–1.038)	0.115	1.003 (0.980–1.026)	0.825	1.003 (0.980–1.026)	0.810
LDL	1.012 (1.006–1.017)	< 0.001	1.010 (1.004–1.015)	0.001	1.011 (1.006–1.017)	< 0.001	1.012 (1.006–1.018)	< 0.001

*Note:* ^a^Model 1: Crude; Model 2: Adjusted for age and gender; Model 3: Further adjustments for smoking, drinking alcohol, physical activity, and BMI; Model 4: Further adjustments for calorie intake. Odds ratios (ORs) represent the change in odds of CVD per unit increase in energy‐adjusted intake (g/day).

Abbreviations: HDL, high‐density lipoprotein; LDL, low‐density lipoprotein; TG, triglyceride.

Table 4 presents the associations between cardiovascular diseases (CVDs) and dietary fat intake across four logistic regression models. In Model 1, cholesterol intake was significantly associated with increased odds of CVDs (OR = 1.003; 95% CI: 1.000–1.006; *p* = 0.028). However, this association was attenuated and became non‐significant in subsequent models: Model 2 (OR = 1.002; 95% CI: 0.999–1.005; *p* = 0.201), Model 3 (OR = 1.002; 95% CI: 0.999–1.005; *p* = 0.158), and Model 4 (OR = 1.002; 95% CI: 0.999–1.005; *p* = 0.134).

**TABLE 4:** Association between cardiovascular disease (CVD) and energy‐adjusted dietary fat intake (g/day) across four logistic regression models.

	Model 1		Model 2		Model 3		Model 4	
	OR (95% CI)	*p*	OR (95% CI)	*p*	OR (95% CI)	*p*	OR (95% CI)	*p*
Cholesterol	1.003 (1.000–1.006)	0.028	1.002 (0.999–1.005)	0.201	1.002 (0.999–1.005)	0.158	1.002 (0.999–1.006)	0.134
Trans fatty acid	2.062 (0.858–4.955)	0.106	1.519 (0.619–3.729)	0.362	1.600 (0.635–4.030)	0.319	1.717 (0.669–4.404)	0.261
SFA	1.011 (0.970–1.054)	0.607	1.026 (0.983–1.071)	0.236	1.036 (0.990–1.084)	0.123	1.042 (0.994–1.091)	0.086
MUFA	0.931 (0.867–0.998)	0.045	0.922 (0.857–0.993)	0.032	0.901 (0.834–0.974)	0.009	0.895 (0.827–0.969)	0.006
PUFA	1.056 (0.977–1.141)	0.170	1.017 (0.937–1.103)	0.690	1.042 (0.955–1.136)	0.359	1.069 (0.966–1.183)	0.199
Omega‐3	0.863 (0.089–1.292)	0.344	0.115 (0.035–2.763)	0.239	0.449 (0.054–4.846)	0.421	0.332 (0.071–3.807)	0.446
DHA	0.347 (0.000–482.216)	0.157	0.327 (0.043–2.062)	0.141	0.734 (0.014–1.352)	0.256	0.343 (0.084–2.501)	0.276
EPA	1.229 (0.351–4.308)	0.748	1.575 (0.418–5.942)	0.502	1.643 (0.424–6.375)	0.473	1.481 (0.375–5.849)	0.575
ALA	0.971 (0.929–1.015)	0.191	0.977 (0.932–1.023)	0.321	0.974 (0.928–1.022)	0.280	0.975 (0.929–1.024)	0.309
Omega‐6	0.958 (0.851–1.078)	0.478	0.954 (0.845–1.078)	0.455	0.945 (0.834–1.071)	0.377	0.942 (0.831–1.067)	0.346

*Note:* Model 1: Crude; Model 2: Adjusted for age and gender; Model 3: Further adjustments for smoking, drinking alcohol, physical activity, and BMI; Model 4: Further adjustments for calorie intake.

Abbreviations: ALA, alpha‐linolenic acid; DHA, docosahexaenoic acid; EPA, eicosapentaenoic acid; MUFA, monounsaturated fatty acid; PUFA, polyunsaturated fatty acid; SFA, saturated fatty acid.

Trans fatty acid intake showed no significant association with CVDs in any model: Model 1 (OR = 2.062; 95% CI: 0.860–4.944; *p* = 0.106) and Model 4 (OR = 1.717; 95% CI: 0.663–4.451; *p* = 0.261). Similarly, saturated fatty acids (SFA) were not significantly associated with CVDs: Model 1 (OR = 1.011; 95% CI: 0.956–1.070; *p* = 0.607) and Model 4 (OR = 1.042; 95% CI: 0.991–1.096; *p* = 0.086). In contrast, MUFA intake was consistently associated with a reduced risk of CVDs across all models: Model 1 (OR = 0.931; 95% CI: 0.869–0.998; *p* = 0.045), Model 2 (OR = 0.922; 95% CI: 0.859–0.990; *p* = 0.032), Model 3 (OR = 0.901; 95% CI: 0.835–0.972; *p* = 0.009), and Model 4 (OR = 0.895; 95% CI: 0.828–0.967; *p* = 0.006). we tested interaction terms between MUFA intake and key covariates (age, sex, BMI, diabetes, and physical activity), and none reached statistical significance. The consistency of associations across strata suggests that the inverse association between MUFA intake and CVD risk is unlikely to be explained by these characteristics. Polyunsaturated fatty acid (PUFA) intake showed no significant association with CVDs in any model: Model 1 (OR = 1.056; 95% CI: 0.972–1.147; *p* = 0.170) and Model 4 (OR = 1.069; 95% CI: 0.957–1.195; *p* = 0.199). Intake of total omega‐3 fatty acids, including DHA, EPA, and ALA, as well as omega‐6 fatty acids, did not show significant associations with CVDs in any of the models (all P‐values > 0.05; 95% CIs included the null value).

## 4 Discussion

This study explored the complex association between cardiovascular diseases (CVDs) and lipid profile with various dietary fats. Our results showed that patients had a significantly higher mean age, weight, and BMI compared to controls, consistent with previous studies indicating that age and obesity are major risk factors for CVDs [23, 24]. These differences in dietary intake suggest potential patterns that may be associated with cardiovascular health, although causal interpretations are limited by the cross‐sectional design. Patients reported notably lower protein intake, total fat, carbohydrates, and total energy, aligning with the theory that inadequate nutrient intake could heighten cardiovascular vulnerability [25]. The lower cholesterol intake among cases may indicate dietary changes after a CVD diagnosis, a typical adaptive behaviour [25, 26].

Notably, the patients had a significantly lower intake of MUFA compared to the controls. Greater consumption of MUFA and PUFA is generally considered to be protective due to their positive effects on lipid metabolism and inflammation [27, 28]. Insufficient intake of these fatty acids may adversely affect cardiovascular health [29, 30]. MUFA may exert cardioprotective effects through multiple mechanisms, including improving lipid profiles, reducing oxidative stress and inflammation, and enhancing endothelial function, thereby lowering the risk of atherosclerosis and cardiovascular events [31]. We also observed a significant positive association between CVDs and serum levels of total cholesterol and LDL, consistent with prior research connecting hypercholesterolemia and elevated LDL levels to an increased risk of cardiovascular events [32, 33]. However, no significant association was found between CVDs and serum triglyceride or HDL levels, which may be explained by the complex interplay of genetic, dietary, and metabolic factors influencing lipid metabolism [34, 35, 36, 37, 38].

The analysis revealed that MUFA intake was consistently linked to a lower risk of CVDs across all models; supporting evidence that greater MUFA intake has protective effects on cardiovascular health through improved lipid profiles and decreased inflammation [39, 40]. In contrast, cholesterol intake was initially linked to an increased risk of CVD in the crude model [41, 42]. However, the significance diminished after adjusting for confounding factors, indicating that other dietary and lifestyle elements may mediate this relationship [43]. The absence of a significant link between the intake of trans fatty acids and SFA with the risk of CVD may result from the relatively low consumption of these fats or dietary changes made after a CVD diagnosis [44, 45]. Similarly, consuming PUFA, omega‐3, and omega‐6 fatty acids did not significantly correlate with CVD risk. This observation underscores the need for additional research to clarify the role of specific fatty acids in cardiovascular health [46, 47].

Given the rising prevalence of cardiovascular diseases and the inconsistent findings regarding the role of dietary fats in CVD risk, this study was conducted to clarify these associations within an Iranian population using data from the PERSIAN cohort. This study is characterised by a substantial sample size and comprehensive dietary and clinical data, which significantly enhances the reliability and generalizability of the findings. Nevertheless, possible limitations encompass the dependence on self‐reported dietary intake, which may be influenced by recall bias. Additionally, the study's cross‐sectional design precludes the establishment of causal relationships between dietary factors and the risk of CVD. Interaction or effect modification generally requires larger samples, especially when subgroup sizes are unequal. However, the lack of significant interactions suggests that the protective effect of MUFA intake on CVD risk may be independent of demographic and metabolic characteristics. Nevertheless, we acknowledge that the current study may have limited power to detect small interaction effects, and larger prospective studies are needed to confirm these findings. In the present analysis, potential confounders were adjusted for based on both theoretical relevance and statistical evaluation. We assessed the linearity assumption for continuous covariates (such as age, BMI, and energy intake) by examining their associations with the log odds of CVD using restricted cubic spline functions. Since no substantial nonlinearity was observed, these variables were modelled as linear terms. We acknowledge that residual confounding and model misspecification cannot be completely ruled out; however, sensitivity analyses using alternative model specifications yielded similar results. Future studies with propensity score weighting or double robust methods are warranted to further validate the robustness of our findings. Moreover, prospective studies incorporating lipidomic and metabolomic profiling could enable biomarker‐based calibration of self‐reported dietary fat intake, thereby improving causal inference and enhancing the reliability of diet–disease associations.

## 5 Conclusion

Our findings emphasise the intricate connection among dietary intake with lipid profiles and CVD risk. Higher intake of MUFA was associated with lower CVD risk. However, given the cross‐sectional nature of the data, these results should be interpreted with caution and not as evidence of a causal protective effect. This highlights the importance of dietary quality and lipid management in relation to CVD risk. Additional longitudinal studies are necessary to validate these findings and investigate the underlying mechanisms that drive these associations.

## Author Contributions

N.H.A., K.A.M., S.N., M.S., S.F.N. and S.D. designed the study, were involved in the data collection, analysis, and drafting of the manuscript. S.D., S.M., F.T., PB, M.G., M.A. and A.K. were involved in the design of the study, analysis of the data, and critically reviewed the manuscript. All authors read and approved the final manuscript.

## Funding

The research was funded by Sabzevar University of Medical Sciences, Sabzevar, Iran (Code 99219).

## Ethics Statement

This study was approved by the Ethical Committee of the Research Ethics Committee of the sabzevar University of Medical Sciences, Sabzevar, Iran (code: IR.MEDSAB.REC.1400.021). All procedures of the studies involving human participants were by the ethical standards of the institutional and/or national research committee and the 1964 Declaration of Helsinki and its later amendments or comparable ethical standards.

## Consent

All participants signed informed consent forms. Informed consent was obtained from the adolescents and their parents to participate in the study.

## Conflicts of Interest

The authors declare no conflicts of interest.

## Data Availability

The data that support the findings of this study are available from the corresponding author upon reasonable request.
